# Hypopigmentation in mantled howler monkeys *Alouatta palliata* (gray 1849): First documented cases of whole‐body leucism in South America

**DOI:** 10.1002/ece3.9628

**Published:** 2022-12-08

**Authors:** Cristian Barros‐Diaz, Silvia Vela, Abel Gallo‐Pérez, Manuel Chiquito, Xavier Cornejo, Denis Mosquera‐Muñoz, Julian Perez‐Correa

**Affiliations:** ^1^ Fundación para la Conservación e Investigación JaPu Guayaquil Ecuador; ^2^ Faculdad de Ciencias Exactas y Naturales Pontificia Universidad Católica del Ecuador Pichincha Ecuador; ^3^ Facultad de Ciencias Naturales Universidad de Guayaquil Guayaquil Ecuador; ^4^ Herbario GUAY, Departamento de Botánica, Facultad de Ciencias Naturales Universidad de Guayaquil Guayaquil Ecuador; ^5^ Programa de Maestría en Ciencias Marinas y Costeras, Facultad de Ciencias Naturales Universidad de Guayaquil Guayaquil Ecuador; ^6^ School of Biological Sciences University of Aberdeen Aberdeen UK

**Keywords:** color aberration, connectivity loss, endogamy, equatorial Pacific dry forests, genetic anomaly, pigmentation shift by pollution in primates

## Abstract

The existence of hypopigmentation like leucism is the result of genetic anomalies that might be enhanced by external factors such as pollution. This anomaly may reduce survival rates. Leucism has been recorded in wildlife, but overall, it is considered very rare. There have been few records of mantled howler monkeys with leucism in Mexico and Costa Rica, but whole‐body leucism in howler monkeys from South America was unknown. In this article, we report for the first‐time documented cases of whole‐body leucism in young individuals of mantled howler monkeys *Alouatta palliata* in an isolated remanent of tropical dry forest in southwestern Ecuador known as Cerro Blanco Protective Forest. In total, we found two juvenile individuals with leucism in October 2021. The report of howler monkeys with whole‐body leucism may be caused by two processes: inbreeding because of isolated populations, environmental pressure caused by pollution, or the interaction of both. Our findings also reveal that hypopigmentation is becoming more frequent in howler monkey populations along its distributional range. Therefore, it is important to promote research in this field to disentangle the causes of hypopigmentation and to consider a regional management strategy for the species.

## Graphical abstract

Hypopigmentation in vertebrates is the result of genetic impoverishment and it is associated with isolated populations and environmental stressors. In mantled howler monkeys *Alouatta palliata* (Gray 1849) there are records of hypopigmentation in Mexico and Costa Rica but this is the first documented record of whole‐body leucism in South America in a critically endangered population from a critically endangered ecosystem. Our findings also reveal that hypopigmentation is becoming more frequent in howler monkey's population along its distributional range.

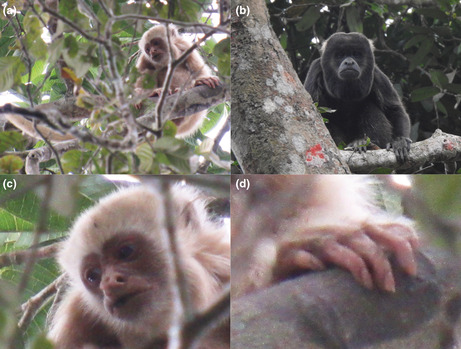

## Abstract (es)

La existencia de afecciones que causan hipopigmentación, como el leucismo, son el resultado de anomalías genéticas que pueden verse potenciadas por factores externos como la contaminación. Estas anomalías puedes reducir las tasas de supervivencia. Se ha registrado leucismo en la vida silvestre, pero en general, se considera muy raro. En México y Costa Rica se ha reportado la existencia de casos aislados de monos aulladores de manto con leucismo, pero se desconocía el leucismo de cuerpo completo en monos aulladores para América del Sur. En este artículo, reportamos por primera vez casos documentados de leucismo en todo el cuerpo en individuos juveniles de monos aulladores de manto *Alouatta palliata* en un remanente aislado de bosque seco tropical en el suroeste de Ecuador conocido como Bosque Protector Cerro Blanco. En total, encontramos dos individuos juveniles con leucismo en octubre de 2021. Este reporte de monos aulladores con leucismo en todo el cuerpo puede ser causado por dos procesos: apareamiento dentro de individuos de la misma población causado por el aislamiento, la presión ambiental causada por la contaminación o la interacción de ambos. Nuestros hallazgos también revelan que la hipopigmentación es cada vez más frecuente en las poblaciones de monos aulladores a lo largo de su rango de distribución. Por lo tanto, es importante promover la investigación en este campo para determinar las causas de la hipopigmentación y considerar una estrategia de manejo regional para la especie.

***Palabras clave:*** aberraciones cromáticas, pérdida de conectividad, anomalías genéticas, endogamia, cambio de pigmentación en primates por contaminación, Bosque Seco del Pacífico Ecuatorial

## 1 INTRODUCTION

Hypopigmentation in wildlife, namely albinism or leucism, is considered a genetic anomaly derived from recessive alleles (Hu et al., 2011; Prado‐Martinez et al., 2013). Albinism is controlled by over 18 genes (Bridge et al., 2014; Hu et al., 2011; Montoliu et al., 2014; Montoliu & Kelsh, 2014; Summers, 2009) and is characterized by the complete loss of pigmentation (Sandoval‐Castillo et al., 2006; Uieda, 2000). On the other hand, leucism is controlled by six genes (Reissmann & Ludwig, 2013); leucistic animals show partial or total loss of pigmentation in skin or fur but maintain normal coloration in the eyes or hands (García‐Morales et al., 2012; Liu et al., 2019; Silva‐Caballero et al., 2014). Vertebrates with these color aberrations are mostly reported in small and isolated populations, where inbreeding causes recessive alleles to be expressed (Bensch et al., 2000). Survival rates of these animals are usually reported to be low (Owen & Skimmings, 1992).

Even though hypopigmentation is mainly gene driven, it is known that environmental stressors might enhance its occurrence. For example, some environmental aspects that are related to hypopigmentation are altered habitats (Kehas et al., 2005), diet (Coimbra‐Filho & da Cruz Rocha, 1978; Peles et al., 1995) and pollution (Martin et al., 2022; Møller & Mousseau, 2001). Vertebrates color depend on two melanin molecules: eumelanin (polymers of indole units that promotes dark and gray colors) and pheomelanin (oligomers of sulfur‐containing heterocycles promoting yellowish, reddish, chestnut and brown colors), produced by the melanocytes (Toral et al., 2008). Both molecules are present in all vertebrates and its proportion varies depending on the prevalence of the color patter of each species (Galvan & Solano, 2009). Besides the genetic control, the exposure to oxidative stress in the environment may shift the proportion of melanin molecules or even inhibit melanin production (Pang et al., 2014; Roulin et al., 2008), deriving in hypopigmentation.

In mammals, hypopigmentation conditions like leucism have been recorded in several taxa, for example, canids (López‐González, 2011), erinaceids (Morris & Tutt, 1996), mustelids (Tortato & Althoff, 2007), pinnipeds (Acevedo & Aguayo, 2008), procyonids (Silva‐Caballero et al., 2014), chiropterans (García‐Morales et al., 2012), rodents (Mejía Valenzuela, 2019), soricids (Guevara et al., 2011), and ursids (Ritland et al., 2001). In neotropical primates, there are several records of abnormal pigmentation in the coat (López‐Platas et al., 2021), for example, Brown howler monkey *Alouatta guariba clamitans* (Aximoff & Vaz, 2016; Fortes & Bicca‐Marques, 2008), Black‐handed spider monkey *Ateles geoffroyi* (Espinal et al., 2016), Black‐tufted marmoset *Callithrix penicillata*, Common Marmoset *Callithrix jacchus*, and their hybrids (Aximoff et al., 2020; do Vale et al., 2018).

The mantled howler monkey *Alouatta palliata* is a neotropical primate distributed from Mexico to Peru. Its global population is considered Vulnerable according to the IUCN Red List (Cortes‐Ortíz et al., 2021). Meanwhile, in Ecuador, local populations are considered Critically Endangered (Tirira, 2021). This species is heavily affected by habitat fragmentation and loss, which reduces habitat quality and increases isolation (Arroyo‐Rodríguez & Mandujano, 2006) that may cause abnormal coloration due to recessive alleles being more likely to be expressed (Aximoff & Vaz, 2016; Fortes & Bicca‐Marques, 2008).

Hypopigmentation in individuals of *A. palliata* has been reported previously in Mexico and Costa Rica (Galván et al., 2019; López‐Platas et al., 2021; Ramos‐Luna et al., 2022; Sánchez‐Soto, 2018), but no record has been found in South America. In this nature note, to our knowledge, we present the first published report of mantled howler monkeys showing whole‐body leucism in South America. In total, we found two juvenile individuals in a private protected forest known as Cerro Blanco Protective Forest (CBPF) located in Guayaquil, Ecuador. We also hypothesized possible causes of this previously unrecorded phenomena.

## 2 METHODS

### 2.1 Description of site

The tropical dry forest is considered critically endangered worldwide (Ferrer‐Paris et al., 2019). Locally, the ecosystem has lost over 70% of its original vegetation (Best & Kessler, 1995; BirdLife International, 2019; Portillo‐Quintero & Sánchez‐Azofeifa, 2010) and less than 3% is protected (Portillo‐Quintero & Sánchez‐Azofeifa, 2010).

**FIGURE 1:**
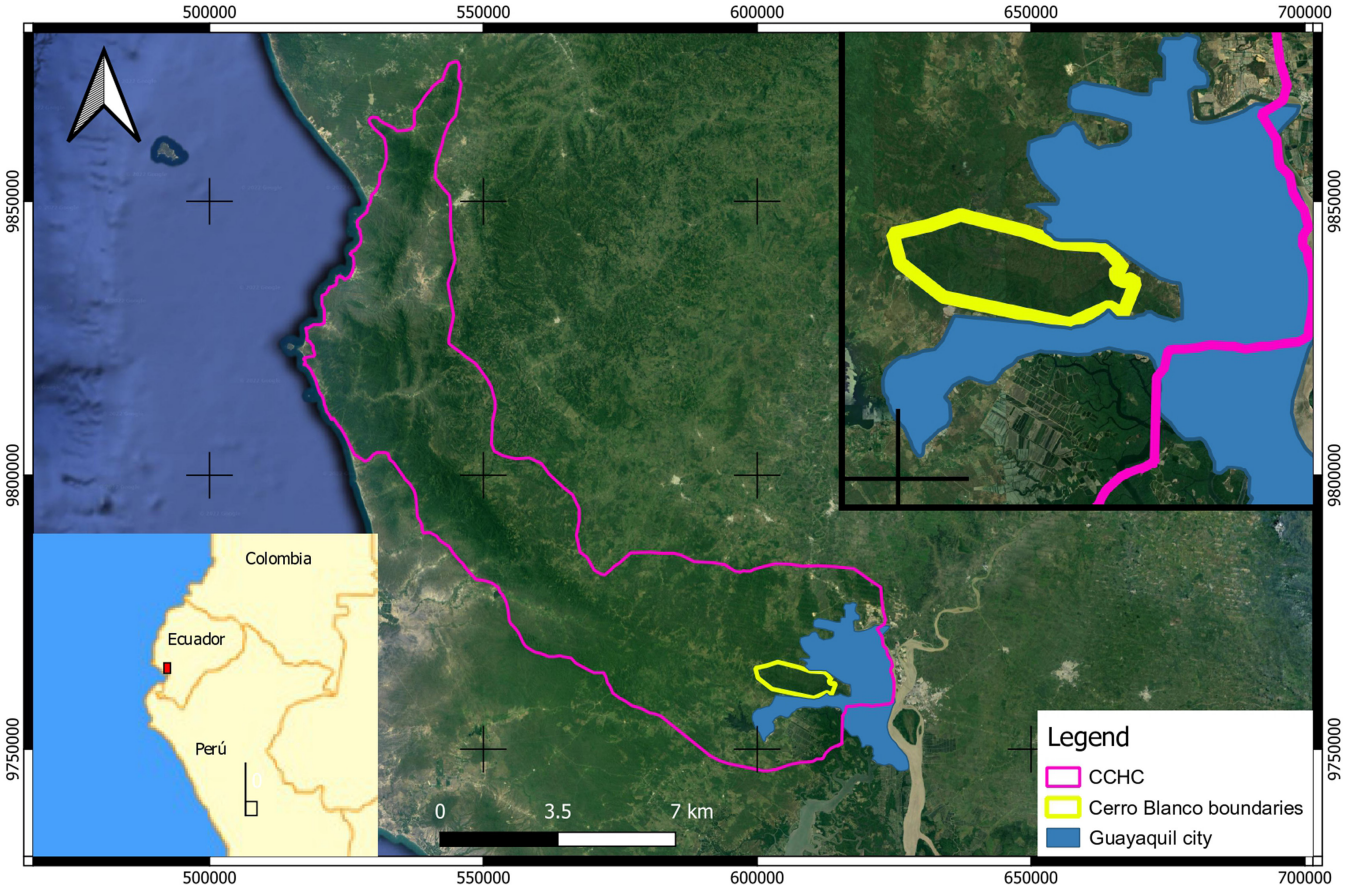
Location of the Cerro Blanco protective Forest (yellow line) with respect to the city of Guayaquil (blue fill) and the Chongon‐Colonche Mountain range (purple line).

Cerro Blanco Protective Forest (CBPF) is a private protected tropical dry forest located in western Guayaquil, Ecuador with a surface of ~6000 ha. The central coordinates of CBPF are 608516.39 E and 9761934.82 S (Figure 1). The area is classed as semi‐deciduous forest transitioning to dry and moist forest, influenced by the absence of rains between June and December. During these months, CBPF only receives horizontal precipitation in the form of a fine seasonal drizzle. The upper canopy reaches 30 m high and is mostly closed or semi‐closed. This reduces the strong impact of sunlight that is characteristic of equatorial dry forest ecosystems. At the same time, the canopy generates a cool microclimate within the sub‐canopy, favorable for vertebrates' occurrence.

**FIGURE 2:**
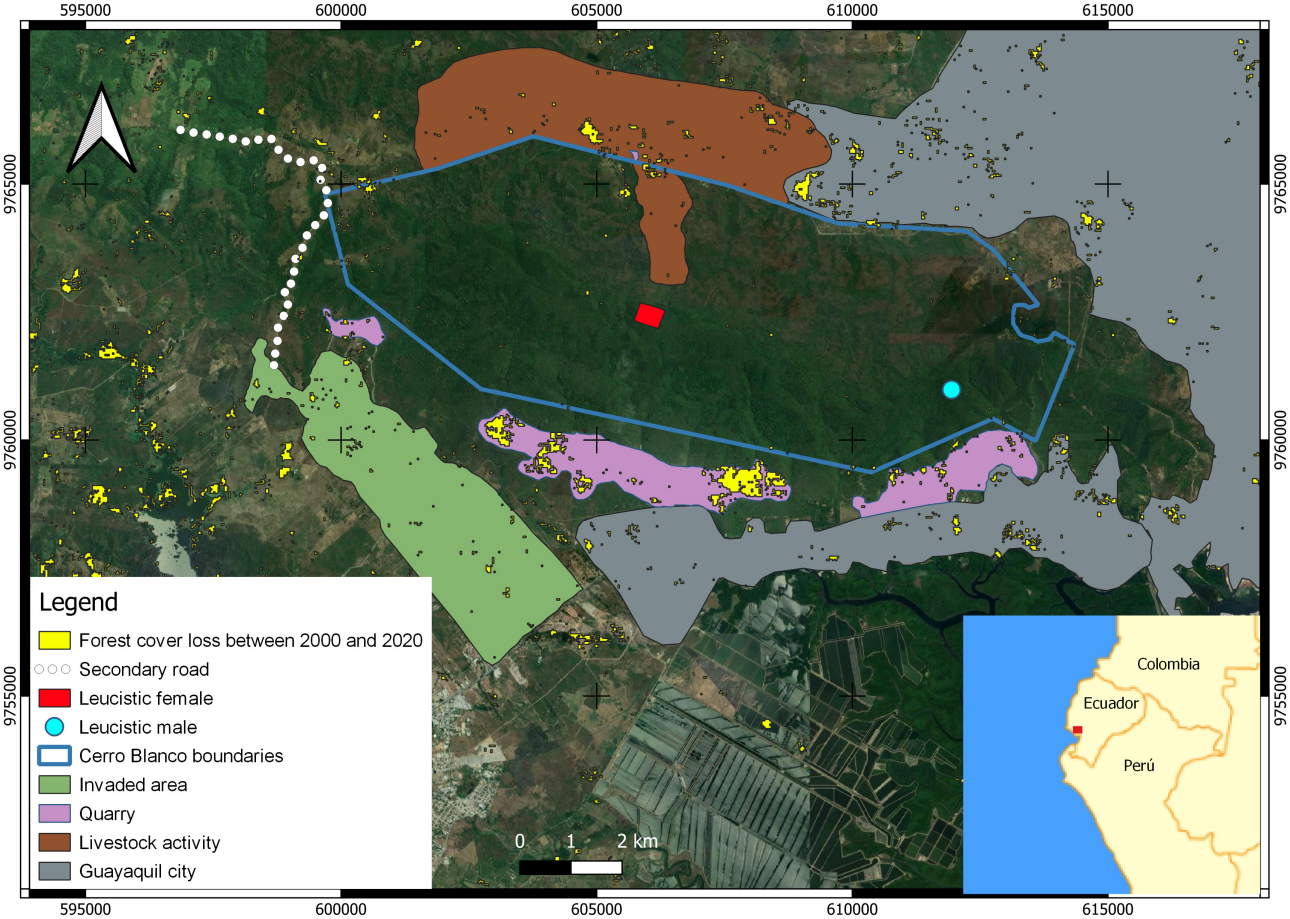
Geographical location of CBPF and current activities that surround the protected and buffer area. Location of leucistic individuals is depicted in polygon shapes (red square for the first individual and light‐blue circle for the second individual).

The CBPF is part of the National Forest Heritage of Ecuador as well as the Chongon‐Colonche Mountain Range. Nevertheless, it is physically surrounded by the city of Guayaquil where occurs several anthropogenic activities and a dramatic change in land use is evident. Human activities nearby CBPF include livestock activities, quarries of aggregates and stone, and urban areas such as the expansion of formal and informal populated areas (human invasions). These activities contribute to environmental pollution. For example, there are records of critical loads of sulfur exceeding permissible levels in mangroves (Quevedo et al., 2018) less than 10 km away from CBPF. On the other hand, Morales‐Estupiñan et al. (2020) report high levels of heavy metal bioaccumulation in plants next to high‐traffic roads. The incidence of human activities in the city at the edge of CBPF remarks on the importance of the area as an isolated refuge for biodiversity with a high loss of connectivity with other nearby protected areas of the Chongon‐Colonche Mountain Range (Figure 2).

### 2.2 Description of mantled howler monkey (*Alouatta palliata*)

Mantled howler is a relatively big monkey with black whole‐body coloration except for the flanks that may be yellowish, golden, or dull brown (Tirira, 2017). It is a diurnal, gregarious, and arboreal primate (Emmons & Feer, 1999) that feeds primarily on leaves. Nevertheless, it may also ingest fruits, nectar, and flowers (Crockett, 1998). Occasionally, insects may be consumed while ingesting plants (Tirira, 2008). The mantled howler monkey lives in groups usually from 2 to 18 members but in well‐preserved forests, groups may be composed of more than 40 individuals. (Emmons & Feer, 1999; Tirira, 2017). Groups consist of one or more adult males, adult females, and young males and females (Chapman & Balcomb, 1998). Both young males and females are separated from the group before adulthood and live in solitary until they are able to join another group (Tirira, 2017).

### 2.3 Observational methods

We observed the individuals opportunistically while doing research on rodents, plants, and birds. Therefore, no systematic method was applied.

## 3 RESULTS

### 3.1 Observation of the first whole‐body leucistic individual

**FIGURE 3:**
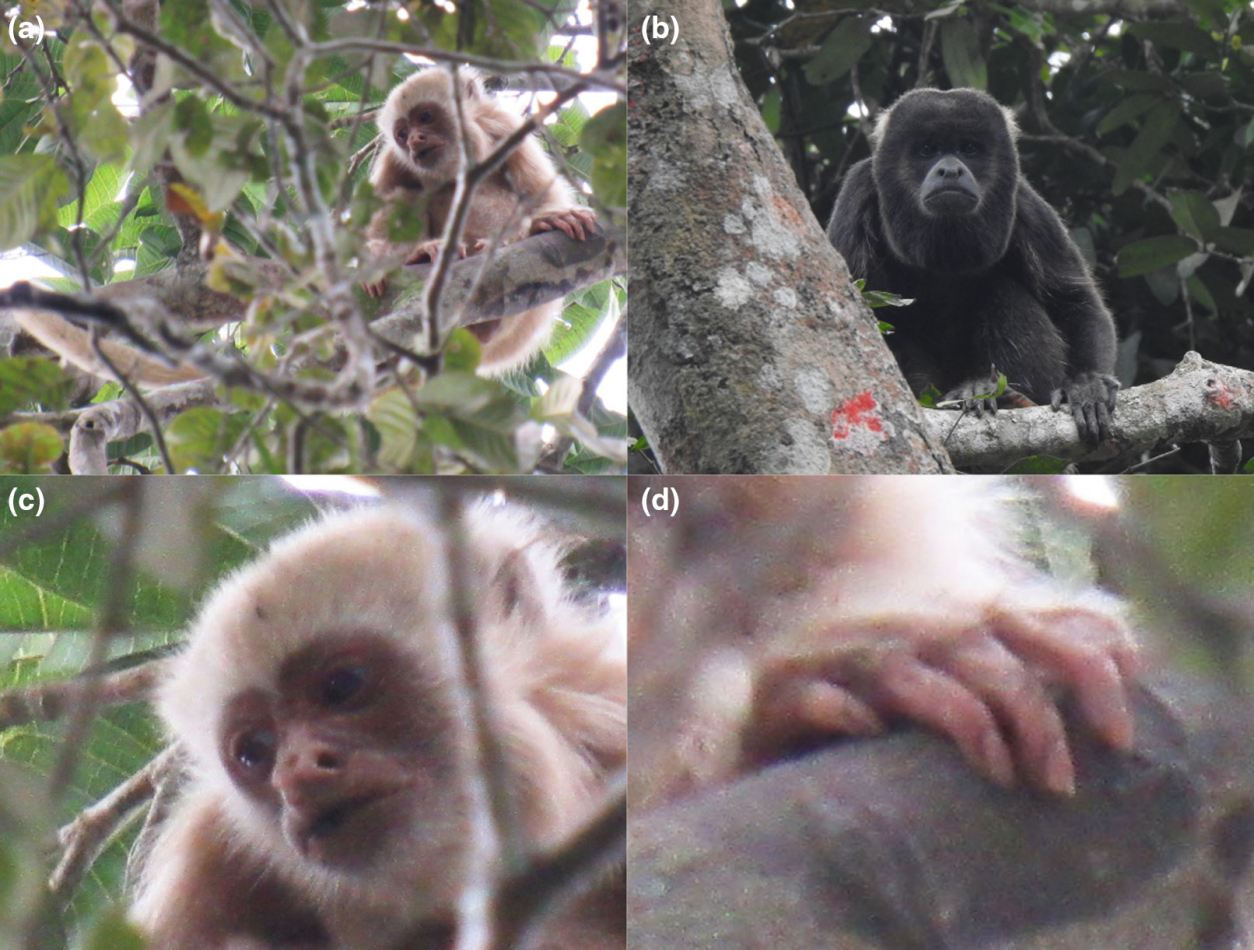
(a) Photograph of the first juvenile individual showing whole‐body leucism located in the canopy of the tree. (b) Photograph of an adult in the group nearby individual sighting showing normal color patterns. Close‐up of individuals' head showing normal dark pigmentation on eyes (c) and left nails showing decoloration (d). (photos a, c, d, by Xavier Cornejo; b, by Julian Perez‐Correa).

We made the first observation of the young individual with whole‐body leucism on October 11, 2021, at 4:50 p.m. EST at 606199 E ‐ 9762444 S. We subsequently spotted the individual on October 12 (10:55 a.m.) and 14 (4:00 p.m.) not far from the first site. The observations lasted between 2 and 8 min. Initially, we identify a group of 14 howler monkeys at approximately 10 m high. The group was composed of juveniles and adults. Nearby the highest tree, we noticed a juvenile howler monkey with whole‐body hypopigmentation showing a pale‐cream fur except for eyes, that remained with dark pigmentation. (Figure 3). In all observations, the individual was at least 5 meters apart from the group.

### 3.2 Observation of the second whole‐body leucistic individual

**FIGURE 4:**
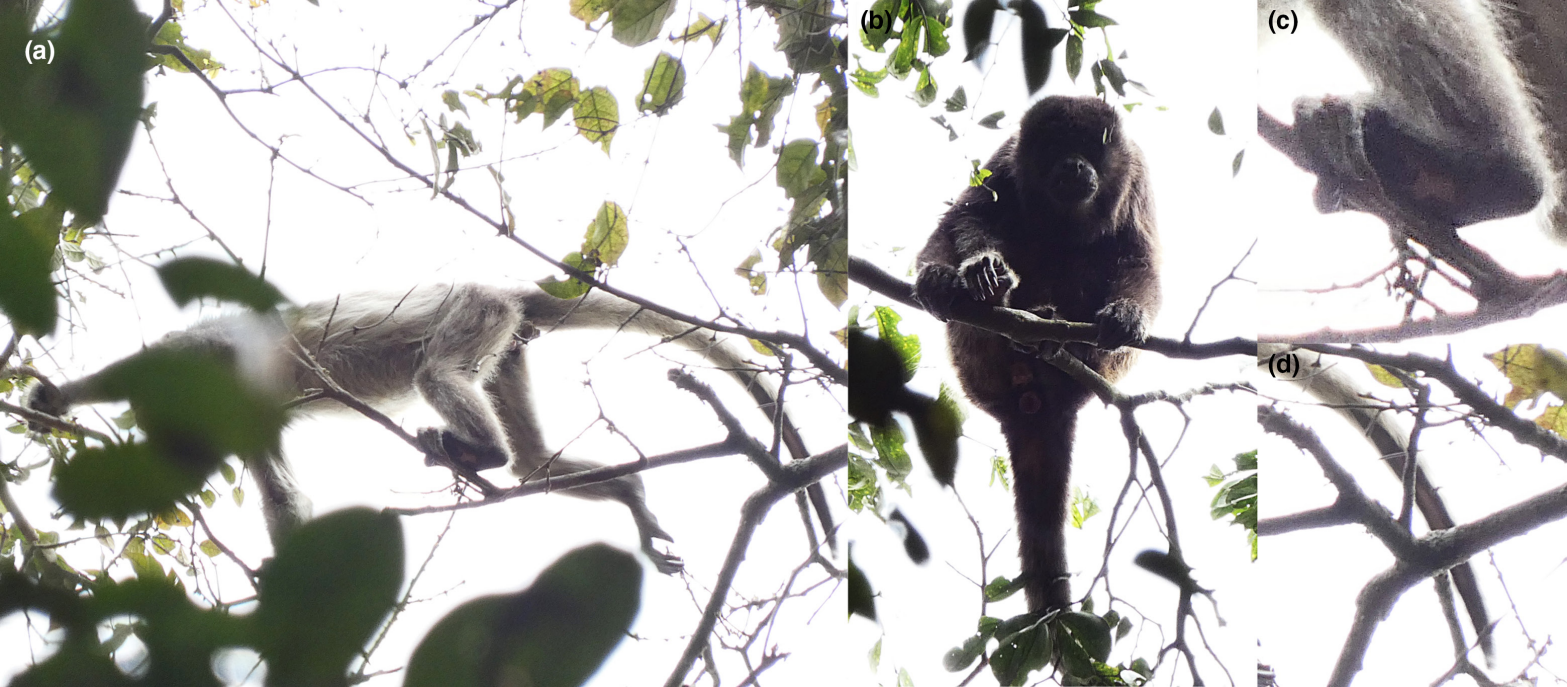
(a) Photograph of the second individual with whole‐body leucism. (b) Photograph of an adult in the group nearby individual sighting showing normal color patterns. Close‐up of individuals' tail (c) and feet (d) showing normal pigmentation on the palm (c) (photos a, b, c, d by Denis Mosquera).

We observed the second whole‐body leucistic juvenile only once, on November 11th, 2021 at 8:42 a.m. EST for ~3 min at 612212 E ‐ 9760699 S. It was part of a group of seven individuals (composed of 2 juveniles and 5 adults) at approximately 14 meters high. There was a small chance to observe the individual and take photos because most of the time it stayed between the higher branches, as we described for first leucistic individual (Figure 4). The individual presented a white‐to‐gray pigmentation in its fur with dark‐spotted palms and tail.

## 4 DISCUSSION

Hypopigmentation, like leucism, occurs with low frequency in wild animals (Acosta, 2005; Lopez‐Ortega & Carbo‐Ramirez, 2010; Møller et al., 2013) and its particularly rare in primates (Aximoff & Vaz, 2016; Espinal et al., 2016). Individuals of *A. palliata* with hypopigmentation have been reported previously in Mexico and Costa Rica. Sánchez‐Soto (2018) and Galván et al. (2019) reported evidence of partial decoloration in tail or arms. Meanwhile, Ramos‐Luna et al. (2022) reported whole‐body leucism in anecdotal data from social media (i.e. Facebook) and López‐Platas et al. (2021) reported a case of whole‐body piebaldism in *A. palliata* subspecies *mexicana*. In this manuscript, we report the observation of two juvenile mantled howler monkeys showing partial loss of pigmentation, maintaining dark tones in the eyes and hands. Hence, we think the individuals are leucistic. There are no records of similar color aberrations within at least 1000 km away of any population of *A. palliata*. Therefore, the isolated appearance of this phenomenon has occurred independently within an endangered population (Tirira, 2021) in an endangered ecosystem (Ferrer‐Paris et al., 2019). Our discovery should be seen as possible alarm of broken genetic flow between unconnected populations of *A. palliata*.

Cerro Blanco protective forest is under strong pressure due to the constant growth of the city of Guayaquil and its surroundings, limestone quarries, agriculture, and livestock. These activities cause isolation from the rest of the Chongon‐Colonche Mountain Range. In addition, CBPF experiences pollution generated by the city, mining, and farming activities which may induce a change in color patterns in primates (Galván et al., 2019). At the stage of this research, we can only speculate about the drivers of hypopigmentation shown by mantled howler monkeys in CBPF. Three hypotheses derive from our observations: (1) the appearance of these whole‐body leucism is related to isolation of population, (2) the appearance of leucism might be the result of long‐time exposure to environmental pollution, and (3) furthermore, we speculated that both drivers might be interacting causing hypopigmentation in *A. palliata* population in CBPF.

With respect to our first hypothesis, it is very likely that the expression of recessive genes in these whole‐body leucistic individuals may be the result of a lack of genetic exchange due to isolation, where the inbreeding process is possibly increasing the population's homozygous condition. This hypothesis coincides with the loss of alleles reported by Dunn et al. (2014) for the Mexican howler monkey. However, there are other cases of isolated populations of *A. palliata* in similar systems (e.g., Jauneche, Los Ríos, Ecuador) but there are no confirmed cases of similar phenomena.

Pollution may exacerbate the appearance of hypopigmentation in vertebrates. For example, Martin et al. (2022) found that the long‐time exposure to heavy metal changed the skin pigmentation in the checkerboard worm lizard (*Trogonophis wiegmanni* Kaup, 1830). On the other hand, in a in vivo experiment, Plonka et al. (2006) demonstrate that the ingestion of zinc sulfate caused the inhibition of eumelanin production in mice. In howler monkeys, Galván et al. (2019) described a pigmentation shift from eumelanin to phaeomelanin possibly as a result of the ingestion of leaves contaminated with sulfur‐based pesticides, suggesting that sulfur is added to melanin molecules resulting in phaeomelanin. In this manuscript, we also hypothesized that the independent and simultaneous occurrence of two juvenile howler monkey with leucism in two separate groups might be also a result of exposure to pollution. In Guayaquil, there is evidence of high levels of sulfur (Quevedo et al., 2018) and heavy metals such as Zinc, Copper, Magnesium, Nickel and Chrome (Morales‐Estupiñan et al., 2020). It is possible that one of these pollutants is promoting changes in melanogenesis pathways producing leucism in howler monkeys in Guayaquil. However, as this is not the scope of the research and the encounters were merely anecdotal, we are not able to confirm the causes of the phenomena.

In addition, we think that there might be an interaction between genetic drivers and environmental stressors that are causing hypopigmentation. It is known that oxidative stress caused by external agents such as pollution produces DNA damage directly affecting melanogenesis (Chen et al., 2021). In the case of the juveniles howler monkeys reported in this manuscript, the continuous presence of pollutants such as heavy metals and sulfur (Morales‐Estupiñan et al., 2020; Quevedo et al., 2018) might cause oxidative stress in howler monkey individuals and possible other vertebrated, promoting pigmentation changes, similar to what has been hypothesized by Galván et al. (2019) in Costa Rica. We further think that the occurrence of this phenomena in the area is likely to increase in the next few years as the area surrounding CBPF is considered an area of urban expansion.

To our knowledge, the observation of the individuals represents the first documented cases of whole‐body leucism for mantled howler monkeys in South America. This information contributes to what has been found in Mexico and Costa Rica (Galván et al., 2019; López‐Platas et al., 2021; Ramos‐Luna et al., 2022; Sánchez‐Soto, 2018). Although our hypotheses are to an extent, speculative, as we were not able to capture any individuals to make further analysis (e.g., genetic, hair analysis), it is remarkable the importance to promote research in these fields to disentangle the causes of hypopigmentation (genetic pressure, environmental pressure, or the interaction of both) and to understand the effects of this phenomena over ecological and social behavior of the mantled howler monkeys. As a final recommendation, it is also very relevant to set regional strategies for the conservation of the mantled howler monkey that is considered vulnerable worldwide (Cortes‐Ortíz et al., 2021) and critically endangered in Ecuador (Tirira, 2021).

## AUTHOR CONTRIBUTIONS

**Cristian Barros‐Diaz:** Conceptualization (lead); formal analysis (lead); funding acquisition (equal); investigation (equal); methodology (equal); project administration (supporting); supervision (supporting); validation (equal); visualization (equal); writing – original draft (lead); writing – review and editing (supporting). **Silvia Vela:** Investigation (equal); methodology (equal); writing – review and editing (supporting). **Abel Gallo‐Perez:** Conceptualization (supporting); investigation (equal); methodology (equal); project administration (supporting); writing – review and editing (supporting). **Manuel Chiquito:** Investigation (supporting); methodology (supporting); writing – review and editing (supporting). **Xavier Cornejo:** Conceptualization (equal); investigation (equal); validation (equal); writing – original draft (equal); writing – review and editing (equal). **Denis Mosquera‐Muñoz:** Conceptualization (equal); formal analysis (lead); investigation (supporting); methodology (supporting); supervision (supporting); writing – original draft (supporting); writing – review and editing (equal). **Julian Perez‐Correa:** Conceptualization (lead); formal analysis (equal); funding acquisition (lead); investigation (equal); methodology (supporting); project administration (lead); supervision (lead); validation (lead); visualization (equal); writing – original draft (lead); writing – review and editing (lead).

## Supporting information


Video S1.

## Data Availability

Data sharing is not applicable to this article as no datasets were generated or analysed during the current study
